# Preface: special topic on natural products and drug discovery

**DOI:** 10.1093/nsr/nwac244

**Published:** 2022-11-02

**Authors:** Zhen Yang

**Affiliations:** Zhen Yang School of Chemical Biology and Biotechnology, Peking University Shenzhen Graduate School, China

During the past few decades, several FDA-approved medicines inspired by natural products have reached the clinic. The time is therefore right to publish a special topic dedicated to the topic of natural products and drug discovery. This special topic contains one review article, four perspectives, two research highlights, two research articles and a personal interview. We have invited a collection of recognized experts in the field of natural product chemistry to contribute to this special topic, which provides a broad survey of progress over the past few years.

The special topic starts with an interesting and detailed review by David J. Newman, who discusses historically important discoveries regarding the therapeutic use of natural products as anti-infective, antitumor and antimetabolic agents. Some of these are currently widely used as first-line drugs or have provided the starting point for developing much better semi-synthetic analogs for the treatment of infectious diseases, cancer and metabolic diseases.

In accordance with the majority of research in this area, the four perspectives presented by Geoffrey A. Cordell, Jian-Min Yue, Hui Ming Ge and Xiaoyu Tang are devoted to describing recent progress in sustainable strategy development, and the isolation, synthesis and biosynthesis of biologically active natural products by using emerging tools and techniques. Geoffrey A. Cordell discusses the topic ‘Evolving paradigms for natural-product drug discovery’ and presents his long-standing desire to integrate the technologies of the Fourth Industrial Revolution (4IR) with sustainability in biologically active natural products to meet global human health-care needs. Bin Zhou and Jian-Min Yue present a perspective entitled ‘Natural products are the treasure pool for antimalarial agents’ and highlight the historical achievements and current trends in antimalarial drug discovery based on natural products. In the perspective contributed by Bo Zhang and Hui Ming Ge, the authors discuss state-of-the-art pericyclases for cycloaddition and outline the issues associated with, and opportunities for, further studies of pericyclases. The perspective entitled ‘Harnessing nature's biosynthetic capacity to facilitate total synthesis’ by Xiaoyu Tang and coworkers highlights representative applications of synthetic biology in the total syntheses of complex molecules by harnessing the power of biosynthetic enzymes.

In the research highlight section, Tuoping Luo discusses the technology developed by Ian B. Seiple and coworkers in which total synthesis is combined with cryo-electron microscopy to resolve complexes of virginiamycin derivatives with the *Escherichia coli* 50S ribosome. The author focuses on an integrated approach that facilitates the identification of new and potent antibiotics with reduced bacterial resistance. The highlight contributed by Ziyang Zhang, entitled ‘Beyond natural targets: chemical synthesis reprograms the target specificity of rapamycin’, describes a compelling strategy developed by Jun O. Liu and coworkers, which shows that modification of natural products can not only expand the target landscape of natural products, but also impart improved properties such as potency and selectivity to the parent molecules.

This special topic also contains two research articles, by Xiaoguang Lei's group and Zhen Yang's group, on drug discovery based on natural products. Xiaoguang Lei's group designed and synthesized bifunctional chemical probes based on the natural product polycarcin V as site-specific DNA-damaging agents, and uncovered novel DNA–protein interactions. This highlights their potential use in the light-controlled treatment of skin diseases such as psoriasis. Zhen Yang's group reported their efforts to achieve the isolation and semi-synthesis of the natural product (−)-anisomelic acid from *Anisomeles indica* (L) Kuntze (Labiatae) leaves, which has been identified as an oral agent against SARS-CoV-2 in mice.

In the final section of the special topic, Hongkui Deng summarizes the efforts of his group to use small-molecule natural products for *in vitro* generation of many pluripotent stem cells from patients. Such cells have the potential to be used for cell therapy, drug screening and disease modeling, and are the most important ‘seed cells’ in the field of regenerative medicine.

This special topic provides information on research in the areas of natural products and drug discovery. We hope that this five-part publication, which is devoted to demonstrating that natural products are a primary source of medicines, reflects current state-of-the-art drug discovery, and will become a useful reference both for beginners and veterans in this dynamically developing field.

## Notes

Guest Editor of Special Topic

Editorial Board Member of NSR

